# Airfall volume of the 15 January 2022 eruption of Hunga volcano estimated from ocean color changes

**DOI:** 10.1007/s00445-024-01744-6

**Published:** 2024-05-29

**Authors:** Liam J. Kelly, Kristen E. Fauria, Michael Manga, Shane J. Cronin, Folauhola Helina Latu’ila, Joali Paredes-Mariño, Tushar Mittal, Ralf Bennartz

**Affiliations:** 1 Department of Earth and Environmental Sciences, Vanderbilt University, Nashville, TN USA; 2 Department of Earth and Planetary Science, University of California, Berkeley, Berkeley, CA USA; 3 School of Environment, University of Auckland, Auckland, New Zealand; 4 Tonga Geological Services, Ministry of Natural Resources, Nuku’alofa, Tonga; 5 Department of Geosciences, Penn State University, State College, PA USA

**Keywords:** Hunga volcano, Explosive eruption, Ash volume, Ocean color, MODIS, Submarine volcano, Reflectance

## Abstract

On 15 January 2022, Hunga volcano erupted, creating an extensive and high-reaching umbrella cloud over the open ocean, hindering traditional isopach mapping and fallout volume estimation. In MODIS satellite imagery, ocean surface water was discolored around Hunga following the eruption, which we attribute to ash fallout from the umbrella cloud. By relating intensity of ocean discoloration to fall deposit thicknesses in the Kingdom of Tonga, we develop a methodology for estimating airfall volume over the open ocean. Ash thickness measurements from 41 locations are used to fit a linear relationship between ash thickness and ocean reflectance. This produces a minimum airfall volume estimate of ${1.8}_{-0.4}^{+0.3}$ km^3^. The whole eruption produced > 6.3 km^3^ of uncompacted pyroclastic material on the seafloor and a caldera volume change of 6 km^3^ DRE. Our fall estimates are consistent with the interpretation that most of the seafloor deposits were emplaced by gravity currents rather than fall deposits. Our proposed method does not account for the largest grain sizes, so is thus a minimum estimate. However, this new ocean-discoloration method provides an airfall volume estimate consistent with other independent measures of the plume and is thus effective for rapidly estimating fallout volumes in future volcanic eruptions over oceans.

**Supplementary Information:**

The online version contains supplementary material available at 10.1007/s00445-024-01744-6.

## Introduction

The 15 January 2022 eruption of Hunga volcano in the Kingdom of Tonga was remarkable in part due to its 55–58 km high eruption plume (Carr et al. 2022; Gupta et al. 2022), extensive umbrella clouds (Global Volcanism Program 2022), global impact of atmospheric air pressure waves (Wright et al. 2022), and Pacific wide tsunami (Borrero et al. 2023). Seafloor surveys revealed that at least 6.3 km^3^ (Clare et al. 2023; Seabrook et al. 2023) of material was mobilized and likely deposited by submarine sediment-laden currents. On land, millimeters to centimeters of tephra were deposited and sampled by the authors on several islands throughout the Kingdom of Tonga. Eyewitness reports of tephra deposition were recorded in additional locations, and minor pumice rafts were created (Paredes-Mariño et al. 2023). Here we seek to quantify the total volume of the 15 January 2022 Hunga airfall tephra deposits including both fall deposits on land and over the open ocean. Erupted volume is important for understanding the scale of an eruption, relating it to other historical eruptions and eruptions in the geologic record. This specific eruption has garnered global attention and fueled discussion on the importance of the interaction of external water with volcanic plumes (Witze 2022).

A challenge in estimating the tephra fallout volume from the 15 January 2022 Hunga eruption is that most of the fallout occurred over the open ocean. There have been many other instances of eruptions where large fractions of erupted tephra were deposited over the ocean (e.g., Toba, Rose & Chesner 1987; the Aleutians, Westberry et al. 2019), and this has caused issues when estimating eruptive volume for eruptions such as Pinatubo 1991 (Paladio-Melosantos et al. 1996) or Hudson volcano 1991 (Scasso et al. 1994). In both cases, total tephra volumes were estimated by extrapolating thickness versus isopach area plots that were generated with land-based thickness measurements. In some cases, volume estimates can be enhanced by utilizing oceanographic sediment cores (Paladio-Melosantos et al. 1996). In the case of Hunga volcano, however, there is relatively little nearby land area (< 800 km^2^) over which to measure fallout thickness and relatively few seafloor sediment cores to date (e.g., Clare et al. 2023). To overcome this challenge, we combine measurements of tephra thickness across the Kingdom of Tonga with satellite imagery that shows substantial water discoloration around Hunga following the 15 January 2022 eruption.

**Fig. 1:**
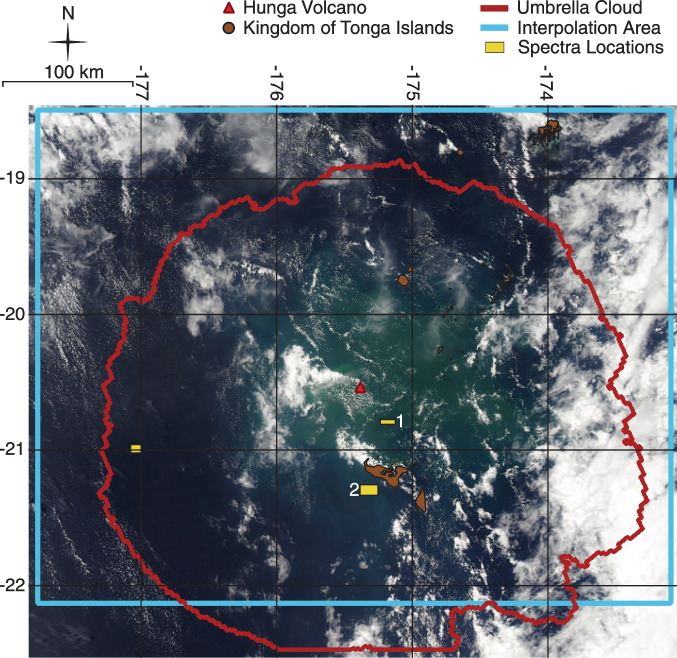
Discoloration around Hunga volcano on 17 January 2022 01:42 UTC shown with outline of umbrella cloud (red) on 15 January 2022 04:50 UTC. Discoloration is seen in the center of the image around Hunga volcano. Islands of the Kingdom of Tonga are shown in brown. Umbrella cloud outline created using data from Gupta et al. (2022). Yellow boxes are areas used to create Fig. 3. Blue rectangle is the interpolation area used for masking clouds and interpolation in Google Earth Engine (see “Methods”). Volume is calculated over the whole image, see Fig. 5A and Supplemental Fig. 4 for example. Islands courtesy of Tonga Department of Statistics and OCHA Office of the Pacific Islands

Discolored water was visible in the ocean around Hunga starting on mid-day 17 January 2022 (early 17 January UTC) after the volcanic plume had dissipated enough to allow a clear view by satellites (Fig. 1). We attribute this initial water discoloration primarily to tephra particles within the water, as did Whiteside et al. (2023) who determined that the ocean spectra, light attenuation, and timescale of discoloration formation were in line with discoloration via tephra. A small fraction of the water discoloration may have resulted from indirect ash effects such as phytoplankton blooms stimulated by the tephra (Barone et al. 2022). Here we will use the discoloration of water surrounding Hunga, as quantified using satellite remote sensing reflectance, combined with measurements of thickness of tephra deposits on land, to estimate the total magnitude and distribution of the tephra fallout deposit.

Reflectance is a measure of how much radiance that contacts a surface is reflected upwards off the surface (Mobley 2020). Reflectance generally has two types when considering ocean applications: surface (or irradiance reflectance, used in this study due to its availability on Google Earth Engine) and remote sensing reflectance (commonly Rrs). Their differences lie in the fact that surface reflectance is non-directional, whereas Rrs is a measure of how much of the downwelling radiance incident to the water in any direction is returned in a specific direction (Mobley 2020). To date, reflectance and associated satellite products have been used to characterize volcanic deposits, including characterization of tephra and relative ages of lava flows at Mt. Etna (Spinetti et al. 2009), and investigation of tephra remobilization, redistribution, weathering, and grain sizes at Sunset Crater in Arizona (Hooper & Necsoiu 2014). These studies focused on the spectral characteristics of subaerial volcanic deposits, distinguished characteristics such as grain size, shape, texture, and weathering using reflectance, and utilized spectroradiometers as well as LiDAR to analyze and validate their findings (Hooper & Necsoiu 2014; Spinetti et al. 2009).

Reflectance intensity can also be applied over the open ocean, as has been done to estimate suspended particulate matter (Wei et al. 2021) as well as calculate bio-optical characteristics (Komick et al. 2009; Kritten et al. 2020;  Zheng & DiGiacomo 2017). In general, it has been shown that reflectance increases with concentration of total suspended solids in the ocean (Ritchie et al. 1976). Because volcanic tephra in water is a suspended solid, we expect that reflectance intensity should increase with the concentration of tephra in the upper meters of the water column. MODIS satellite imagery shows that reflectance intensity is enhanced in the water around Hunga in the week following the 15 January 2022 eruption (Fig. 1, Supplemental Fig. 1). We note that reflectance is sensitive to the upper meters of the water column, and this depth is measured by evaluating the attenuation coefficient. The specific depths of attenuation for a wavelength of 490 nm (K_d_(490), between blue and green wavelengths) around Hunga volcano are no greater than 30 m (Whiteside et al. 2023). Therefore, tephra must stay suspended within these depths to be visible by satellites.

Here we use ocean color (more specifically reflectance intensity) as a proxy for tephra suspended in the water column and to estimate tephra fallout volume. To do this, we combine 41 thickness measurements of tephra across the Kingdom of Tonga with multispectral satellite imagery from MODIS. Estimating erupted volume quickly is very important during crisis management because it allows authorities to plan the appropriate response. Our method enables volume estimates where tephra deposition occurs over water and hence is difficult to map and sample directly.

## Methods

### Tephra collection

**Fig. 2:**
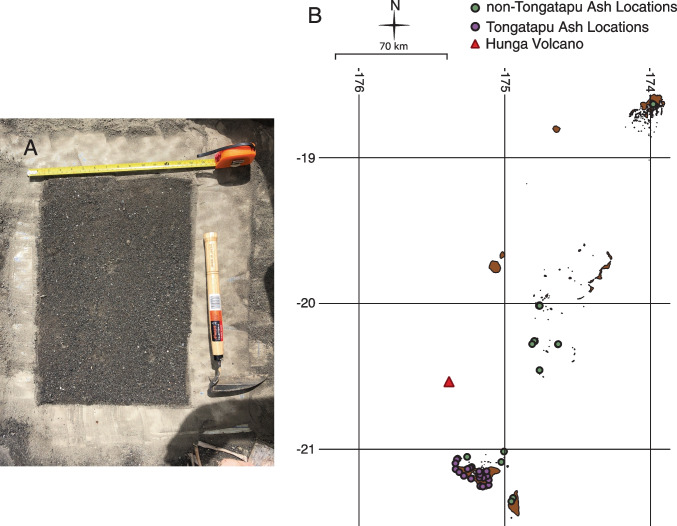
**A** Image of free square of ash brushed and measured for thickness, mass, and area. **B** Sampling locations for ash thickness around the Kingdom of Tonga used in the fitting procedure (Fig. 4). The values of these points are listed in Supplemental Table 1. Islands courtesy of Tonga Department of Statistics and OCHA Office of the Pacific Islands

We collected tephra thickness and surface density measurements throughout the Kingdom of Tonga a few days after the eruption until up to 5 months after the eruption. Flat areas were selected—usually abandoned houses where there were concrete pads that allowed us to either insert a ruler into the deposit to measure tephra thickness and/or collect tephra over a known area with a brush (Fig. 2A). Care was taken to avoid areas with wind or water redistribution or disturbance by human, vehicle, or animal activities. In all cases, multiple locations were measured and sampled in each area to ensure reproducibility. Variability in measured areas was on the order of 10% based on measurements of up to 10 flat-lying and undisturbed sites within an area of ~ 1 km^2^, and < 5% when measuring up to 10 different spots on a single flat concrete foundation or flat roof area of ~ 50 m^2^. We dried and weighed the collected ash samples to calculate surface density (mass/area). We used tephra thickness measurements to calibrate reflectance measurements. All collected field data, including sampling dates, are available in Supplemental Table 1. Some were sampled shortly after the eruption, but the majority were sampled in March of 2022. We also provide in Supplemental Table 2 some preliminary information on grain sizes. Samples were mechanically dry-sieved at half-*φ* intervals (*φ* =  − log2*D*, where *D* is the particle diameter in millimeters). The fine material (< 1 mm) was analyzed with a laser diffraction instrument (MALVERN Mastersizer 3000). Combining the two data sets was done according to Dinis and Castilho (2012). We refer to the material as ash because the majority of the grain sizes have sizes of 1 mm or less, and we provide ash modes for laboratory-analyzed Hunga ash (Paredes-Mariño et al. 2023; see also Supplemental Table 2).

Ash thickness can be related to surface density if the ash sample bulk density is known such that ${\rho }_{B}=M/(A\times H)$, where ${\rho }_{B}$ is the sample bulk density, *M* is the dry sample mass, *A* is the field collection area, and *H* is the ash thickness. We calculated the dense-rock equivalent (DRE) volume using the skeletal and bulk densities of collected tephra. We define ${V}_{DRE}={V}_{b}\times {(\rho }_{b}/{\rho }_{s})$, where ${V}_{DRE}$, ${V}_{b}$, ${\rho }_{b}$, and ${\rho }_{s}$ are the DRE volume, bulk volume (details of the calculation in the “Volume calculation” section), bulk density of tephra, and skeletal density of tephra. We also note that the porosity of the system can be defined as $1-\phi ={\rho }_{b}/{\rho }_{s}$. We measured ash skeletal densities using a Micrometrics AccuPyc II 1340 Gas-Pycnometer utilizing nitrogen at University of Auckland. In addition, we report bulk densities measured in the lab by weighing and loosely packing dry Hunga ash in a cylinder of known volume (Supplemental Table 1).

### Volume calculation

Our method for estimating eruption volume is analogous to methods that integrate isopach maps of ash thicknesses (Pyle 1989). Here, however, rather than measuring deposit thickness directly, we relate the spectral intensity (reflectance) of ocean water surrounding Hunga to measurements of ash thickness on land to create an isopach map. We can then plot deposit thickness versus isopach area and integrate the reflectance-derived isopach map to obtain a total tephra volume.

**Fig. 3:**
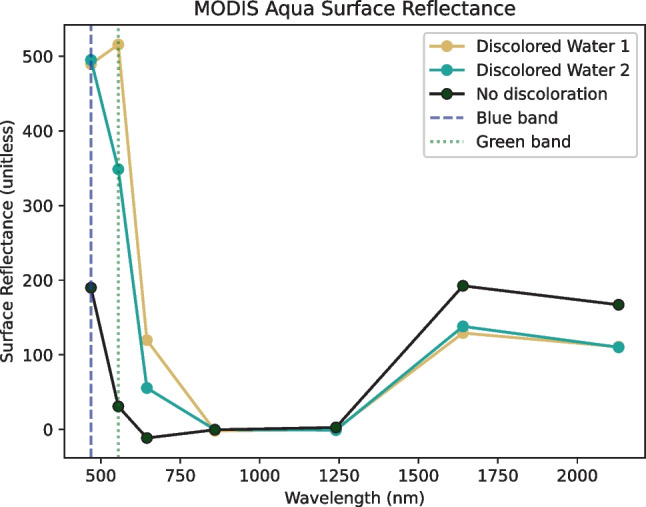
Spectra plot of MODIS Aqua bands 1–7 on 17 January 2022. All three curves correspond to a yellow box in Fig. 1. The two discolored water legend entries are areas in the discolored region in Fig. 1. The area of no discoloration is taken in a darker-colored area from Fig. 1, the westernmost yellow box. See also Supplemental Fig. 2

To obtain quantitative information on ocean discoloration, we used satellite imagery from the instrument MODIS onboard NASA’s Aqua satellite, which provides data in the visible and infrared spectral bands at 250, 500, or 1000 m resolution, depending on the band. We performed image processing and analysis in Google Earth Engine (GEE; Gorelick et al. 2017) and we specifically used “MYD09GA.061 Aqua Surface Reflectance Daily Global 1 km and 500 m” (Vermote & Wolfe 2015). Through inspection of spectra in the ocean (Fig. 3, see also Supplemental Fig. 2), the main difference between ocean water with and without discoloration was in the green and blue reflectance bands (~ 555 nm and ~ 469 nm, respectively); discolored water has a higher reflectance intensity in the green and blue bands. We therefore chose to average the reflectance value of the blue and the green bands together to create a single reflectance value for each pixel indicative of the degree of water discoloration. This is in line with other ocean products that also utilize wavelengths representative of green and blue wavelengths (e.g., particulate organic carbon and chlorophyll-*a*; Hu et al. 2019; O’Reilly et al. 1998; Stramski et al. 2008).

Before utilizing an image, we classified the type of data present at each pixel into one of four categories (clouds, land, deep ocean, shallow/coastal ocean) using the “state_1km” band (see Fig. 1 and Supplemental Fig. 1 to see the presence of clouds). This classification process was particularly important for identifying and masking clouds and land masses from the imagery. We obtained the non-ocean pixels and used an interpolation function to obtain reflectance values within these areas (interpolation area, see Fig. 1) based on the reflectance values in the surrounding ocean areas. This replaces the masked cloud pixels and land pixels with interpolated pixels for reflectance. Specifically, we used the ee.FeatureCollection.inverseDistance() function in Earth Engine to interpolate our dataset, which calculates the inverse-distance weighted estimate value for each pixel from the global mean and standard deviation. We used an interpolation window of $8\times {10}^{4}$ m, and gamma, which controls how quickly estimates tend toward the global mean, was set to a value of 0.3. Varying gamma from 0 to 1 causes a change in reflectance of up to ~ 4 units (up to 1%). Interpolation windows that are too small do not fully cover the pixels left empty due to cloud masking.

Once an interpolated reflectance image was computed, we could then relate tephra thickness measured at a point on land to a reflectance value in nearby ocean waters. To do this, we utilized ash estimates from 41 locations on 11 different islands within the Kingdom of Tonga (Supplemental Table 1, Fig. 2B). These estimates are split between ash estimates on Tongatapu and ash estimates on other islands. In total, we used 21 points from Tongatapu and 20 points from other islands in our analysis. In the full dataset, there are 49 points from Tongatapu alone (Supplemental Table 1). To avoid heavily skewing our analysis toward points on Tongatapu, 21 points were selected randomly without replacement from ash measurements on Tongatapu. Exactly which points used in the analysis are indicated in Supplemental Table 1.

To determine an ocean reflectance value that reflected the conditions close to a land-based tephra measurement, we averaged reflectance values within polygons of ~ 73 km^2^ (~ 300 pixels) around each tephra point measurement (Supplemental Fig. 3). As a result, each tephra thickness value has a corresponding reflectance value.

**Fig. 4:**
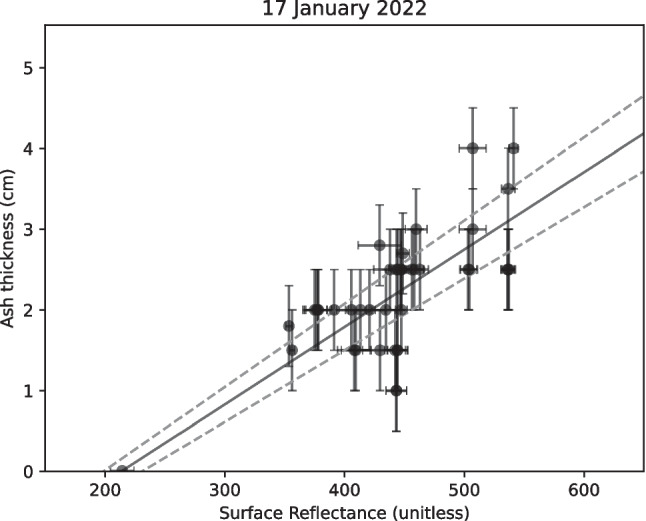
Examples of fits used to calculate eruptive volumes. Solid line is the least-squares best-fit. Dashed lines show the linear fit for *c* (Eq. 1) with margin of error added or subtracted from *c*. Error bars in reflectance are one standard deviation from the mean of average reflectances from Google Earth Engine. Errors bars in ash thickness are based on field measurements. *R*^2^ value is 0.95, degrees of freedom is 39 (*n* = 41 samples − 2), and *t*-score is ~ 2.023. *t*-Scores are calculated using the scipy.stats.t.ppf() function for a two-tailed distribution

**Fig. 5:**
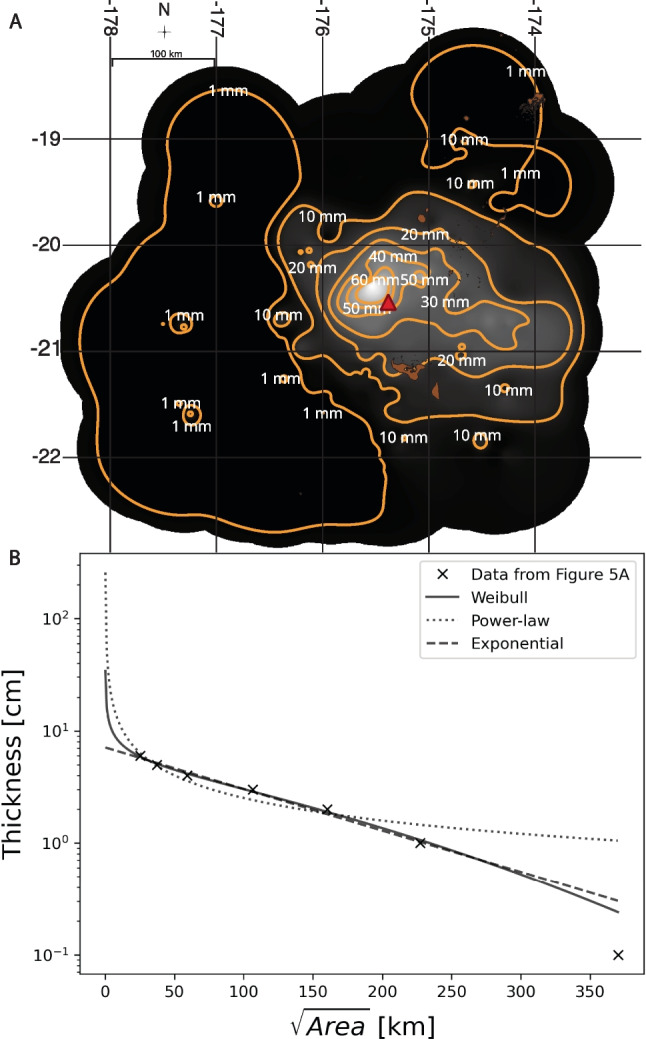
Representation of our results utilizing traditional tephra dispersion methods. **A** Isopach map showing computed ash thickness using the method presented in this manuscript with image showing intensity of reflectance/ash thickness. We see that highest thicknesses are closest to Hunga volcano (red triangle). Furthest from the volcanic vent, we see lower ash concentrations. Contour intervals are spaced every 10 mm. Isopach map morphology suggests source from the lower umbrella cloud of this eruption (Gupta et al. 2022). **B** Thickness vs. square root of isopach area plot. Weibull (solid), power-law (dotted), and exponential (dashed) lines of best fit are shown in relation to the data. Each data point is the area of the isopachs shown in **A**. Volume estimate for the Weibull fit, power-law fit, and exponential fit were calculated following Bonnadonna and Costa (2012, 2013) and Bonadonna and Houghton (2005). Weibull fit gives 1.8 km^3^, exponential fit gives 1.0 km^3^, and the power-law fit gives a volume of 0.2–0.8 km^3^, depending on the integration limits. All three functions were fit using scipy.optimize.curve_fit() in python. The Weibull and power-law functions operate under the assumption that there are larger thicknesses closer to the vent, whereas the exponential does not capture that possibility. The power-law function, however, strays from the observed data further from the vent. Islands courtesy of Tonga Department of Statistics and OCHA Office of the Pacific Islands

We assess MODIS imagery from 17, 18, and 22 January 2022 (UTC) for reflectance intensity. For each satellite image, we plot measured tephra thickness versus the local spectral intensity of discolored water. We observe that ash thickness increases generally as a function of surface reflectance (Fig. 4). We choose to fit a linear function to the ash thickness *h* and spectral intensity *R* to calibrate our spectral intensity measurements:

1 $$h=\left(c\pm e\right)R+b$$

forcing these fits through the minimum value of ash thickness measured in this study, where $c,e$, and $b$ are the calculated slope that relates reflectance to thickness, the margin of error of the slope parameter, and the y-intercept, respectively. There is associated uncertainty with reflectance values as well as ash thicknesses. Standard deviations for the reflectance are minor (< 5% for almost all points); we assume that uncertainties from both reflectance and ash thickness are accommodated by considering the margin of error of the slope coefficient. The residuals of our least squares fitting procedure are assumed to estimate the uncertainty in ash thickness measurements and reflectance. Margin of error for linear regression is calculated as the *t*-score value of a system with a given *R*-squared and degrees of freedom multiplied by the standard error of the slope coefficient: $e={t}^{*}*se$. An example of the fits for 17 January 2022 is shown in Fig. 4. See Fig. 5A for an example of an image created by this procedure. We use the best-fit slope to calculate the mean ash volume, and the margin of error of the slope coefficient to estimate the uncertainty.

We use the ash thickness measurement from Neiafu on the island of Vava’u (0.01 cm, see Supplemental Table 1) to approximate no ash thickness, rather than arbitrarily choosing a location where the presence of ash is unknown. Using the LINEST() function in Excel, we generated a linear best fit for all points used in our analysis. We find that *R*^2^ for 17 January is ~ 0.95; thus, we are confident in our use of a linear relation. Using this procedure, we assume the calibration is valid for higher deposit thicknesses outside of those we have measured. We also acknowledge that having only one 0.01 cm thickness measurement and fitting through that point can bias our results. Accompanying excel sheets containing this data are available on Zenodo.

We choose to fit a linear function instead of another type of function (e.g., exponential) to our dataset for three primary reasons. First, reflectance generally increases monotonically with ash thickness in our data. We also lack evidence for a more complex relationship between reflectance and tephra fall, so we choose the simplest regression to do a first-order estimation. Also, for each day, a linear regression without fitting through the point on Vava’u consistently resulted in higher *R*^2^ values than the respective exponential regression.

Once linear calibrations are established for each day, we convert reflectance values to ash thickness throughout each image. The results are volcanic ash isopach maps. We sum ash thicknesses over the whole image and multiply by the surface area of that image, generating total tephra volumes. For each day, the minimum, average, and maximum volumes are estimated from each of our predicted fits. Different days are utilized due to differences in spectral characteristics of the ocean across days, possibly due to the movement of the ocean, sun positioning, waviness, or sinking of ash. The ash thicknesses do not change for the different days because sampling was not done on each day.

Figure 5A shows an isopach map computed with the built-in Contour function in QGIS. We used the results from this procedure to calculate isopach areas for a thickness vs. square root of isopach area plot. This procedure is common for tephra volume estimation and helps to characterize erupted volume by fitting a function to a plot of tephra thinning (Bonadonna & Costa 2012). We compare here three different methods, the Weibull, power-law, and exponential functions, which are methods used in previous calculations of tephra volume (Bonadonna & Costa 2012; Bonadonna & Houghton 2005). It has been shown that the Weibull function is less sensitive to proximal, distal, or missing data (Bonadonna & Costa 2012), so we focus on the Weibull function:

2 $$T=\theta {\left(\frac{x}{\lambda }\right)}^{n-2}{\text{exp}}\left[-{\left(\frac{x}{\lambda }\right)}^{n}\right]$$

where $\theta$, $\lambda$, and $n$ are a thickness scale (cm), characteristic decay length (km), and a shape parameter (dimensionless) (Bonadonna & Costa 2012, 2013). Volume of deposit is calculated by integrating over space (Bonadonna & Costa 2012, 2013):

3 $$V=\frac{2\theta {\lambda }^{2}}{n}$$

We fit the Weibull and exponential functions utilizing scipy.optimize.curve_fit() in python and utilized each corresponding fit to calculate volume. Weibull volume was calculated from Eq. (3), and the exponential and power-law volumes were calculated by solving Eq. (1) in Bonadonna and Costa (2012) and Eq. (6) in Bonadonna and Houghton (2005). Also see the Supplemental Methods for these equations. For the parameters used in these models, see Supplemental Table 3.

## Results

Reflectance intensity generally increases with increasing ash thickness (Fig. 4), although most ash thickness measurements show a limited range of values between 2 and 3 cm. The error bars on ash thickness and reflectance show that, for the most part, the uncertainty in thickness and reflectance are consistent with the margin of error of the slope *c*. We calculate a mean of 1.8 km^3^ airfall volume, a minimum of 1.4 km^3^, and a maximum of 2.1 km^3^ based on 17 January 2022 reflectance values. Fits for reflectance on other days can be found in Supplemental Fig. 5, and plots of volume estimated from reflectance on 17 January and other days are available in Supplemental Fig. 6.

**Fig. 6:**
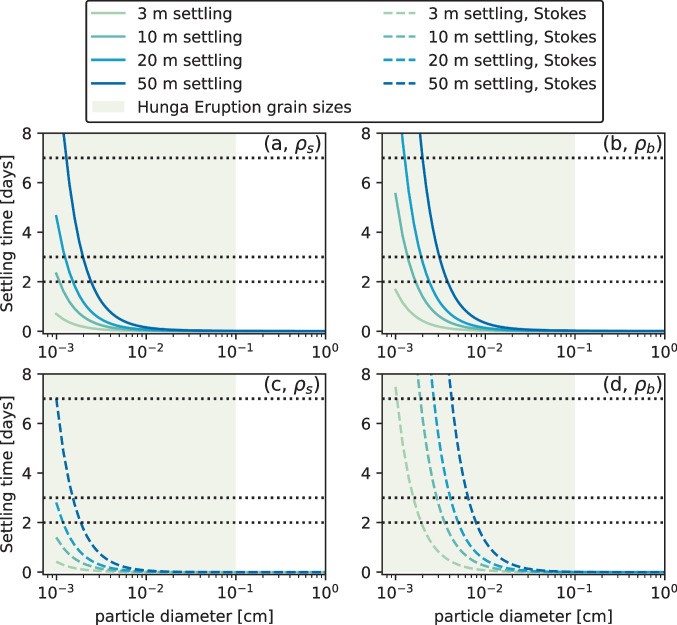
Settling velocities calculated for skeletal (**a**, **c**) and bulk (**b**, **d**) densities of Hunga eruption clasts (2.68 g/cm^3^ and 1.12 g/cm.^3^, see Supplemental Table 2). In all plots, the area in light green indicates the primary particle sizes for the Hunga eruption found by Paredes-Mariño et al. (2023) and shown in Supplemental Table 2. Black lines indicate 17, 18, and 22 January, where 0 days is 15 January 2022, the day of eruption. Settling velocities of finest particles align with our interpretation of the evolution of the signal, as well as the evolution of the attenuation depths found by Whiteside et al. (2023). Settling velocities in **a** and **b** are calculated as sheet-shaped clasts following the procedure outlined in Barreyre et al. (2011). This assumes all of the porosity is connected porosity. Any isolated porosity would increase settling times for all sizes of clasts. Settling velocities in **c** and **d** are calculated using Stokes settling, where we assume particles are perfect spheres. We see that, regardless of the shape of particle, we have persistence of the finest grain sizes in the water column in line with the evolution of penetration depth highlighted by Whiteside et al. (2023)

Bulk densities of ash measured in the laboratory range between 0.968 and 1.166 g/cm^3^. Calculated averages of skeletal density ranging from 2.55 to 2.68 g/cm^3^, the mean of grain size was in the range of 89.1–355 µm, with a large fraction less than 63 µm (Paredes-Mariño et al. 2023; see also Supplemental Table 1 and 2). Utilizing our equation for DRE volume, we calculate mean ${V}_{DRE}$ as 0.75 km^3^ ± 0.03 km^3^, with an accompanying porosity of 56–62%. This value of porosity is in line with other studies of submarine volcanic deposits with porosities around 60% (e.g., Druitt et al. 2024; Walker et al. 1984; Watkins et al. 1978; Wong & Larsen 2010). If instead we utilize bulk densities measured from fall deposits in the field, suggested porosities are between 60 and 80% (Supplemental Table 1), giving a minimum DRE of 0.36 km^3^.

Figure 5A shows our results of generating isopachs from the linear relation between thickness and reflectance. We see that highest thicknesses are closest to the vent (red triangle). As thickness decreases, the appearance of the isopachs becomes less regular. Regardless, we still see a trend of decreasing thickness versus square root of isopach area (Fig. 5B). We show the Weibull, power-law, and exponential best fit lines on the plot. Something to note is that the three functions overlap well within the data, but the Weibull and power-law functions accommodate an assumed increase in thickness closer to the vent, whereas the exponential function does not. However, the power-law function does not fit the data as well as the other 2 functions further from the vent. Volumes calculated are 1.8 km^3^ using the Weibull function, 1.0 km^3^ using the exponential function, and 0.2–0.8 km^3^ using the power-law function.

## Discussion

We estimate a mean airfall volume of 1.8 km^3^ from 17 January imagery and mean airfall volumes of 1.7 km^3^ and 1.4 km^3^ from imagery on 18 and 22 January, respectively. The consistency in airfall estimates suggests that our result is robust to differences in reflectance intensity and ocean color across different days (see Supplemental Fig. 6). The good agreement between our volume calculated from the reflectance image and the volume calculated using the Weibull distribution (Fig. 5B; 1.8 km^3^) highlights that the assumed linear relationship between ash thickness and reflectance is consistent with methods for calculating tephra volume. Our estimated airfall volume is less than the volume of material deposited on the seafloor by density currents, > 6.3 km^3^ (Clare et al. 2023; Seabrook et al. 2023), and > 2.65 km^3^ DRE using the same conversion scaling. Added together, the density current and airfall DRE volumes (~ 3.4 km^3^) are below the estimated volume change of 6 km^3^ DRE estimated by repeat bathymetry analysis at Hunga caldera (Clare et al. 2023; Cronin et al. 2023; Seabrook et al. 2023).

We acknowledge that there are a small number of data points with a narrow range of ash thickness values available to correlate ash and reflectance. These data points are limited by the number of independent ocean island locations where thicknesses can be measured. However, we utilize 41 independent points, with one point for some islands and a total of 21 points on the island of Tongatapu. We also expect tephra settling through the water column to depend on the particle size distribution and density of pyroclasts.

Our estimate of total airfall volume of ${1.8}_{-0.4}^{+0.3}$ km^3^ (mean 0.75 km^3^ ± 0.03 km^3^ of dense-rock equivalent, DRE) represents ~12 % of the caldera volume change (Clare et al. 2023; Cronin et al. 2023; Seabrook et al. 2023). This is consistent with the interpretation that the bulk of the sea-floor deposits were emplaced by gravity currents (Clare et al. 2023; Chaknova et al. 2023) and with recent photographic evidence for a partially collapsing eruptive column (Clare et al. 2023; Fig. S6 within). Using airfall volume alone, the eruption is categorized as VEI 5 on the Volcano Explosivity Index (Newhall & Self 1982). The height of the eruption, reaching the mesosphere (55–58 km, Carr et al. 2022; Gupta et al. 2022; Proud et al. 2022), is greater, however, than historical VEI 5 eruptions such as 1980 Mount St Helens (30 km, Sparks et al. 1986), 1982 El Chichon (32 km, Carey and Sigurdsson 1986), and 2011–2012 Cordón Caulle (14 km, Castro et al. 2013). The great height may be a consequence of the shallow submarine environment that enabled thermal energy from the erupting magma to vaporize water and add to the buoyancy of erupted material (e.g., Fauria et al. 2023; Rowell et al. 2022). Volume estimates from satellite measurements of discoloration would benefit from more examples, observational constraints from deposits on islands and the seafloor, and laboratory experiments to develop and test calibrations and hence the model used to interpret the satellite data.

The full radial extent of the umbrella cloud in Fig. 1 (outline from 04:50 UTC 15 January 2022) is larger than the extent of water discoloration in Fig. 1 (~ 300 km length vs. > 400 km radius). This suggests that much of the fallout occurred within ~ 100 km of the vent and within the spatial and temporal bounds of the umbrella cloud (Fig. 1). Indeed, tephra thicknesses were already thin (~ 0.01 cm) on the island of Vava’u which is > 250 km from Hunga. We note, however, that there is evidence that very fine (< 3 µm) ash was suspended for a longer period in the atmosphere. For example, McKee et al. (2023) observed very fine (< 3 µm) ash within the plume 16 h after eruption onset using the MISR instrument on NASA’s TERRA satellite.

A central assumption in our analysis is that the Hunga ash stays suspended in the upper meters of the water column long enough to be seen by satellites. It is likely some of the Hunga tephra fallout, particularly the largest particles, may have settled below a critical depth before the first MODIS image was taken on 17 January 2022. We estimate settling velocities of ash particles through water and find that, for the finest grain sizes (< 100 µm), it takes on the order of 2 days to settle 10 m and 5 days to settle 20 m in the water column (see Fig. 6; see also Supplemental Methods for equations). We calculate settling times for particles that are sheet-shaped (Fig. 6a, b), as well as perfect spheres that undergo Stokes settling (Fig. 6c, d). We therefore conclude that fine (< 100 µm) particles could have easily stayed suspended within the upper 20 m of the water column until ~ 20 January 2022 (discoloration was visible by satellites until the end of January). Larger particles may have settled earlier (e.g., particles > 100 µm settle in 1 day or less, but the majority of particles on land were less than 100 µm). Generally, however, our calculated total tephra volumes may be considered minimum volumes.

The ability of visible light to penetrate the ocean surface is found by analysis of the attenuation coefficient of light at 490 nm wavelength, K_d_(490) (Whiteside et al. 2023). Higher light attenuation leads to shallower light penetration depths. The settling times of ash particles through the water column are consistent with an observed evolution in penetration depth from 10 m on 17 January to 17 m on 23 January calculated by Whiteside et al. (2023). By early February, penetration depth returned to normal background levels (> 30 m, Whiteside et al. 2023). These estimates of settling velocity that utilize the bulk density potentially include isolated porosity, which only serves to increase these settling times.

We also posit that large particles deposited close to the vent may be underrepresented by ocean color imagery and underestimated here. In general, we lack direct land-based measurements of proximal deposit thicknesses, which impacts volume calculations (Andronico et al. 2014; Klawonn et al. 2014). Despite an expectation of large, and therefore fast settling, particles being deposited closest to the vent, we observe the highest values of reflectance close to the vent. This may indicate that the majority of the Hunga ash was fine (< 1 mm) and/or that the relationship between reflectance and thickness still holds, independent of grain size effects.

It is common for coastal erosion and deposition into the water to cause discoloration (Wei et al. 2021). Reflectance close to the islands can be elevated due to coastal erosion, as was the case in imagery before the 15 January 2022 eruption (Supplemental Fig. 7 shows 30 Dec 2021). On 17 January 2022, however, we do not see elevated reflectance values close to the islands and conclude that coastal erosion is not responsible for the discoloration in Fig. 1. Further, most of the contribution to the volume estimate comes from regions away from coasts. Ocean currents may also have moved the ash from its original location of deposition but are not included in our analyses. Indeed, the elongation of the discolored water patch to the East on 22 January 2022 is possibly due to ocean currents as analyzed by Whiteside et al. (2023). In contrast, the discolored water patch on 17 January 2022 was only slightly elongated to the east and it is likely that the effect of ocean currents is not as strong when compared to later dates.

Barone et al. (2022) argued that the water discoloration on 17 January 2022 included a biologic component from phytoplankton blooms, apparently triggered by ash deposition. Whiteside et al. (2023), however, showed that the optical signature of the Hunga discolored water patch is more consistent with discoloration from inorganic ash particulates. In general, it is common for remote sensing chlorophyll algorithms to produce false positives in particle-rich waters (e.g., Kelly et al. 2023; Komick et al. 2009; Moutzouris-Sidiris & Topouzelis 2021). Either with or without a sudden phytoplankton bloom, a correlation between reflectance intensity and ash thickness may still hold because ash would either have a direct or indirect effect on ocean color. Whiteside et al. (2023) show that the penetration depth of light is greatly impacted by ash in the water column. They make the point that penetration depths are shallower than required for the elevated chlorophyll-*a* concentrations observed by Barone et al. (2022).

The Whiteside et al. (2023) argument that the Hunga water discoloration is from inorganic volcanic ash is convincing, in part, because of the timing of the water discoloration. Phytoplankton blooms do not always occur following ash deposition (Gómez-Letona et al. 2018), and typically take days to develop following the introduction of a limiting nutrient (e.g., Achterberg et al. 2013; Hamme et al. 2010; Langmann et al. 2010). Any chlorophyll present on 17 January 2022 would therefore have resulted from unusually fast growth of phytoplankton. Thus, direct ash deposition from the 15 January 2022 eruption was most likely the primary factor in water discoloration. Barone et al. (2022) suggested that the 13 January 2022 (UTC) Hunga eruption was at least partly responsible for water discoloration and the phytoplankton bloom on 17 January 2022. The 19 December 2021 (UTC) Hunga explosive eruption was similar in column height and magnitude as the 13 January 2022 (UTC) event (Global Volcanism Program 2021, 2022; Gupta et al. 2022; Y. Zheng et al. 2023) but created a much smaller discolored water patch and potential chlorophyll spike to the one observed on 17 January 2022 (Supplemental Fig. 8). As a result, we suggest that any 13 January 2022 effects would be similarly localized to the area adjacent to the volcano. All water discoloration largely dissipated by the end of January/early February 2022 (Barone et al. 2022).

## Conclusions

Our estimate of airfall deposited from the subaerial eruptive plume is at minimum ${1.8}_{-0.4}^{+0.3}$ km^3^, due to the underrepresentation of the largest grain sizes via particle sinking. Mapping of the ocean floor has identified the deposition of > 6.3 km^3^ of new material deposited from sediment-laden currents (Seabrook et al. 2023; Clare et al. 2023) and a caldera volume change of ~ 6 km^3^ DRE (Cronin et al. 2023; Seabrook et al. 2023). This suggests that ~ 12% of the magma volume entered the umbrella region to produce fallout. Overall, this study provides a method based on ocean color to estimate tephra volume over the open ocean, utilizing open source, easily accessed data available on Google Earth Engine. We utilize measured tephra thicknesses at 41 locations (with a large concentration of particles < 63 µm, mostly fine ash) and combine those observations with observations of ocean reflectance to estimate fallout volume. This method can provide a rapid way to estimate erupted volume soon after eruption for ocean volcanoes that have some measurement of deposit thickness on nearby landmasses.

### Supplementary Information

Below is the link to the electronic supplementary material.

Supplementary file1 (DOCX 4456 KB)

Supplementary file2 (XLSX 25 KB)

Supplementary file3 (XLSX 37 KB)

Supplementary file4 (XLSX 11 KB)

## Data Availability

Additional Supporting data is available on Zenodo: 10.5281/zenodo.10420518. The accompanying Google Earth Engine Script for this work is available here: https://code.earthengine.google.com/b93bc807c35fc4af4c8956fc7c9bbb2f. Use requires creation of a free, non-commercial Google Earth Engine account.
