# Supplementary material for: Airfall volume of the 15 January 2022 eruption of Hunga volcano estimated from ocean color changes
Source: Bull Volcanol. 2024 May 29;86(6):59. doi: 10.1007/s00445-024-01744-6 (PMC11136759; doi:10.1007/s00445-024-01744-6)
Supplement: Supplementary file 1 — Supplementary file1 (DOCX 4456 KB) [file 445_2024_1744_MOESM1_ESM.docx]

Bulletin of Volcanology

Supporting Information for

**Airfall volume of the 15 January 2022 eruption of Hunga volcano estimated from ocean color changes**

Liam Kelly^1^, Kristen E. Fauria^1^, Michael Manga^2^, Shane Cronin^3^, Folauhola Helina Latu’ila^4^, Joali Paredes-Mariño^3^, Tushar Mittal^5^, Ralf Bennartz^1^

^1^[lj.kelly@vanderbilt.edu](mailto:lj.kelly@vanderbilt.edu); Department of Earth and Environmental Sciences, Vanderbilt University, Nashville, TN, USA

^2^Department of Earth and Planetary Science, University of California, Berkeley, Berkeley, California, USA

^3^School of Environment, University of Auckland, Auckland, New Zealand

^4^Tonga Geological Services, Nuku’alofa, Ministry of Natural Resources, the Kingdom of Tonga

^5^Department of Geosciences, Penn State University, State College, PA, USA

**Contents of this file**

Supplemental Methods

Supplemental Figures 1-8

Supplemental Tables 1-3

**Introduction**

The supporting information in the supplemental provides a methodology for calculating settling speeds of tephra in the ocean from the Hunga eruption as well as for calculating tephra thicknesses and volumes using the exponential and power-law methods. It also provides different additional figures showing images used to create the isopach map from ocean color, sampling locations, additional fits for imagery on different days, as well as additional information of tephra volumes and settling times for Hunga ash particles. Supplemental Table 1 contains information about sampling, details of which are included in its accompanying metadata file. Supplemental Table 2 contains details of lab measurements of tephra grain size performed at the Unviersity of Auckland. Supplemental Table 3 contains fitted parameters for the Weibull, power-law, and exponential methods of tephra volume calculation. Additional supporting information is available on Zenodo: <https://doi.org/10.5281/zenodo.10420518>.

### Supplemental Methods

#### Tephra Settling

We estimate settling velocities of particles with the density of Hunga particles. To do this, we utilize 2 different equations: Stokes settling and a generalized model put forth by Barreyre et al. (2011). Stokes settling is calculated from

$$v=\frac{2}{9}gR^{2}\frac{\rho_{p}-\rho_{f}}{\mu}$$

where $R, \rho_{p}, \rho_{f}, \mu$ are the radius, density of the particle, density of the fluid, and the fluid viscosity, respectively. Under the assumption of Stokes settling, the particle must be a homogeneous sphere moving through a viscous fluid with very small Reynolds numbers. This does not take into account differently shaped particles, however.

For the consideration of settling of non-spherical particles in the ocean, we turn to Barreyre et al. (2011). They define a generalized settling equation based on samples from different submarine volcanoes. They define settling velocity as

$$w= \frac{\mathrm{Rg}D^{2}}{C_{!}\nu+\left( 0.75C_{2}\mathrm{Rg}D^{3} \right)^{0.5}}$$

where $R$ is submerged specific gravity for clasts in water (dimensionless), $D$ is particle diameter (L), $C_{1}, C_{2}$ are coefficients derived from Stokes Law and asymptotic drag, respectively, $\nu$ is the kinematic viscosity (L^2^T^-1^), $g$ is acceleration due to gravity (LT^-2^), and w is the fall velocity of the particles (LT^-1^). $C_{1}$ and $C_{2}$ are empirically determined coefficients and increase as particles become less spherical. For a perfect sphere, $C_{1}=18, C_{2}=0.4$. We calculated settling velocities for sheet-shaped particles, for which $C_{1}=31.1, C_{2}=14.8$. We utilize 2 different densities for our calculations, the skeletal density (2.68 g/cm^3^) and bulk density (1.12 g/cm^3^) of Hunga clasts which were determined in the lab, see Methods section in main text. Generally, the equation of Barreyre et al. (2011) provides settling velocities slower than traditional Stokes settling, mostly due to particle shape considerations.

#### Tephra Thinning Volume Calculation

In addition to the equations reported in the main text for calculating tephra volume from tephra thinning using the Weibull method, we also utilize the exponential and power-law methods to calculate volume from ash thickness and the square root of isopach area.

Bonadonna & Houghton (2005) define these models, but we will re-state them here. First, the thickness can be calculated for exponential thinning using

$$T=T_{0}e^{-k\sqrt{A}},$$

where $T_{0},k,\sqrt{A}$ are the maximum thickness (cm), the rate of decay (km), and the square root of the isopach area (km).

The definition of thickness as a power-law function is

$$T=T_{pl}\sqrt{A}^{-m},$$

where $T_{pl}$ is a constant (cm/km^-m^) and *m* is the power-law coefficient. Both thickness functions can be used to calculate volume using the following integration

$$V=\int_{0}^{\infty} TdA=\int_{0}^{\infty} xTdx.$$

For the power-law method, the volume can be calculatefd using the simplification

$$V=\frac{2T_{pl}}{2-m}\left( C^{2-m}-B^{2-m} \right),$$

where *B* and *C* are limits of integration, chosen as the location of maximum thickness and the downwind limit of volcanic cloud spreading. For the values of these parameters used in this study, see Supplemental Table 3. Something to note about both of these methods is that the choice of integration limits can greatly impact the calculated volume using the power-law method. We also see that, without higher thickness measurements, the exponential distribution does not accommodate an increase in thickness closer to the vent (Main text, Figure 5b).

## References for Supplemental Material

Barone, B., Letelier, R. M., Rubin, K. H., & Karl, D. M. (2022). *Satellite detection of a massive phytoplankton bloom following the 2022 submarine eruption of the Hunga Tonga-Hunga Haʻapai volcano*.

Barreyre, T., Soule, S. A., & Sohn, R. A. (2011). Dispersal of volcaniclasts during deep-sea eruptions: Settling velocities and entrainment in buoyant seawater plumes. *Journal of Volcanology and Geothermal Research*, *205*(3–4), 84–93. https://doi.org/10.1016/j.jvolgeores.2011.05.006

Bonadonna, C., & Houghton, B. F. (2005). Total grain-size distribution and volume of tephra-fall deposits. *Bulletin of Volcanology*, *67*(5), 441–456. https://doi.org/10.1007/s00445-004-0386-2

## Supplemental Figures


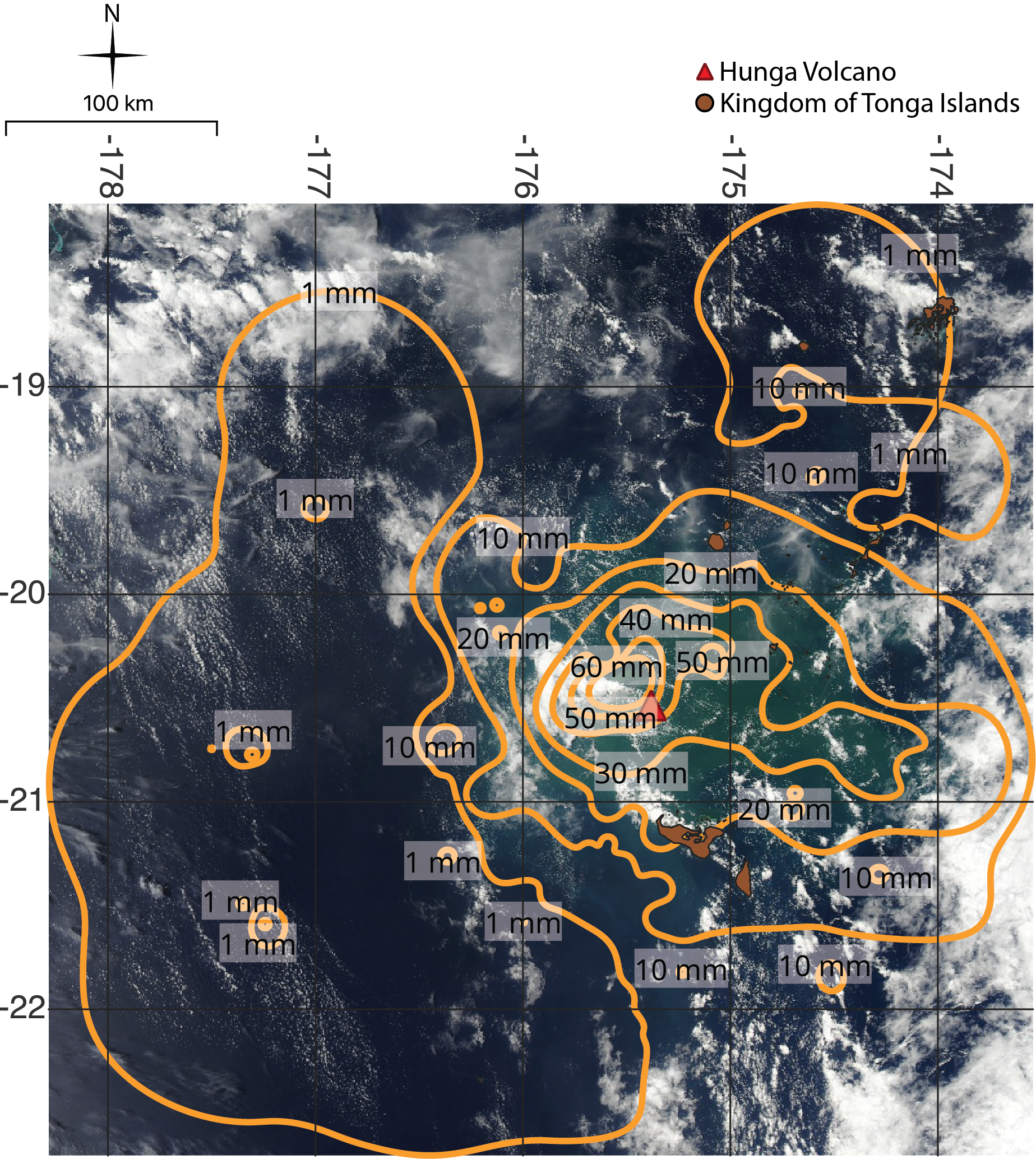


Supplemental Figure 1: Contour map overlain on a truecolor image. Areas where clouds were present and had to be masked. Islands courtesy of Tonga Department of Statistics and OCHA Office of the Pacific Islands.

Supplemental Figure 2: Spectra plot of MODIS Aqua bands 1-7 on 17 January 2022. This plot is an expanded version of Figure 3 in the main text. Here, there are more points for discolored water to show the spread in the blue band. The accompany GEE script used to generate these curves can be found here: <https://code.earthengine.google.com/1c2a827522713c2d8de9f6fda82e59b4>


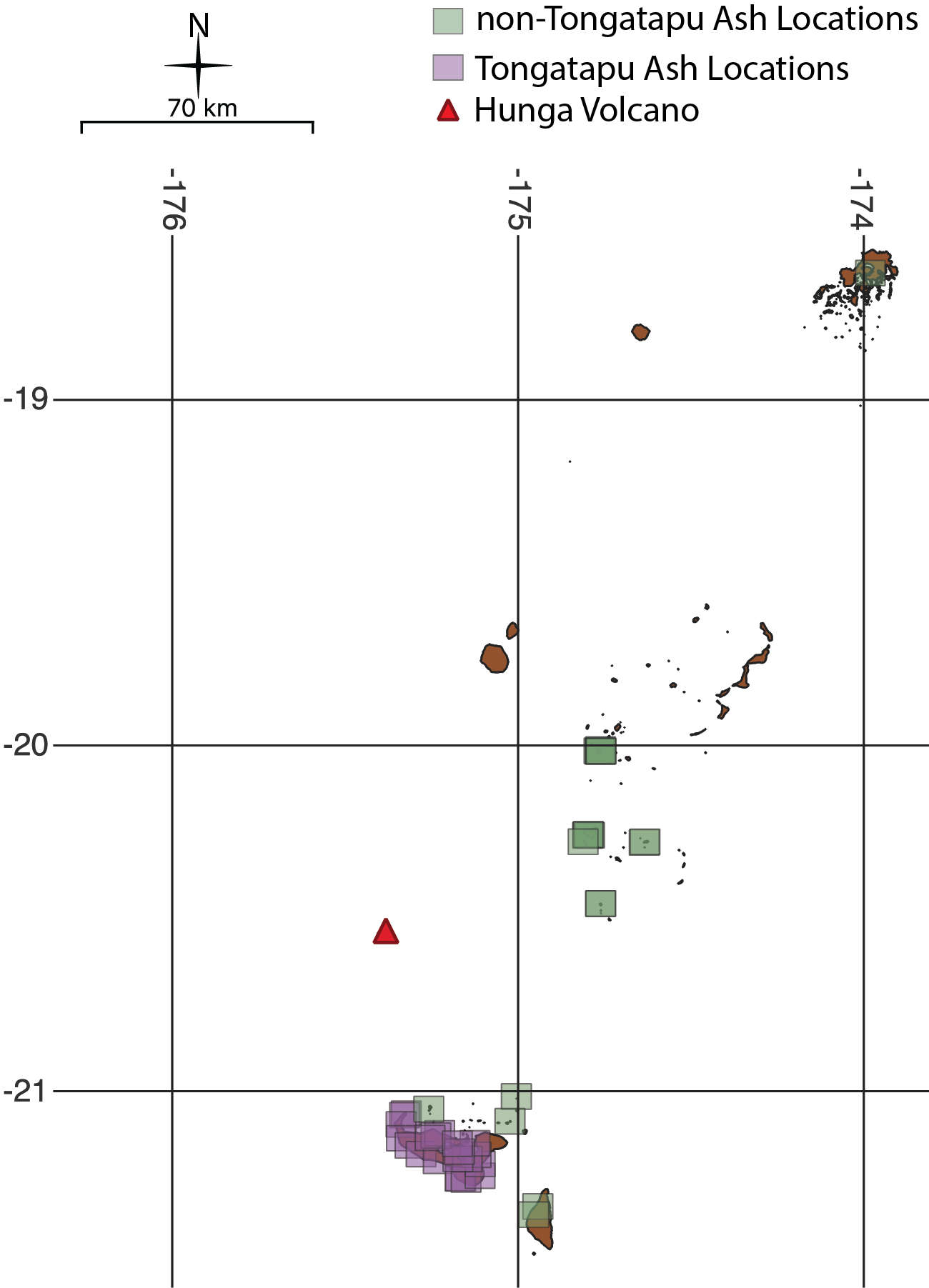


Supplemental Figure 3: Areas over which interpolated reflectance was averaged. Each polygon is ~73 km^2^. Islands courtesy of Tonga Department of Statistics and OCHA Office of the Pacific Islands.


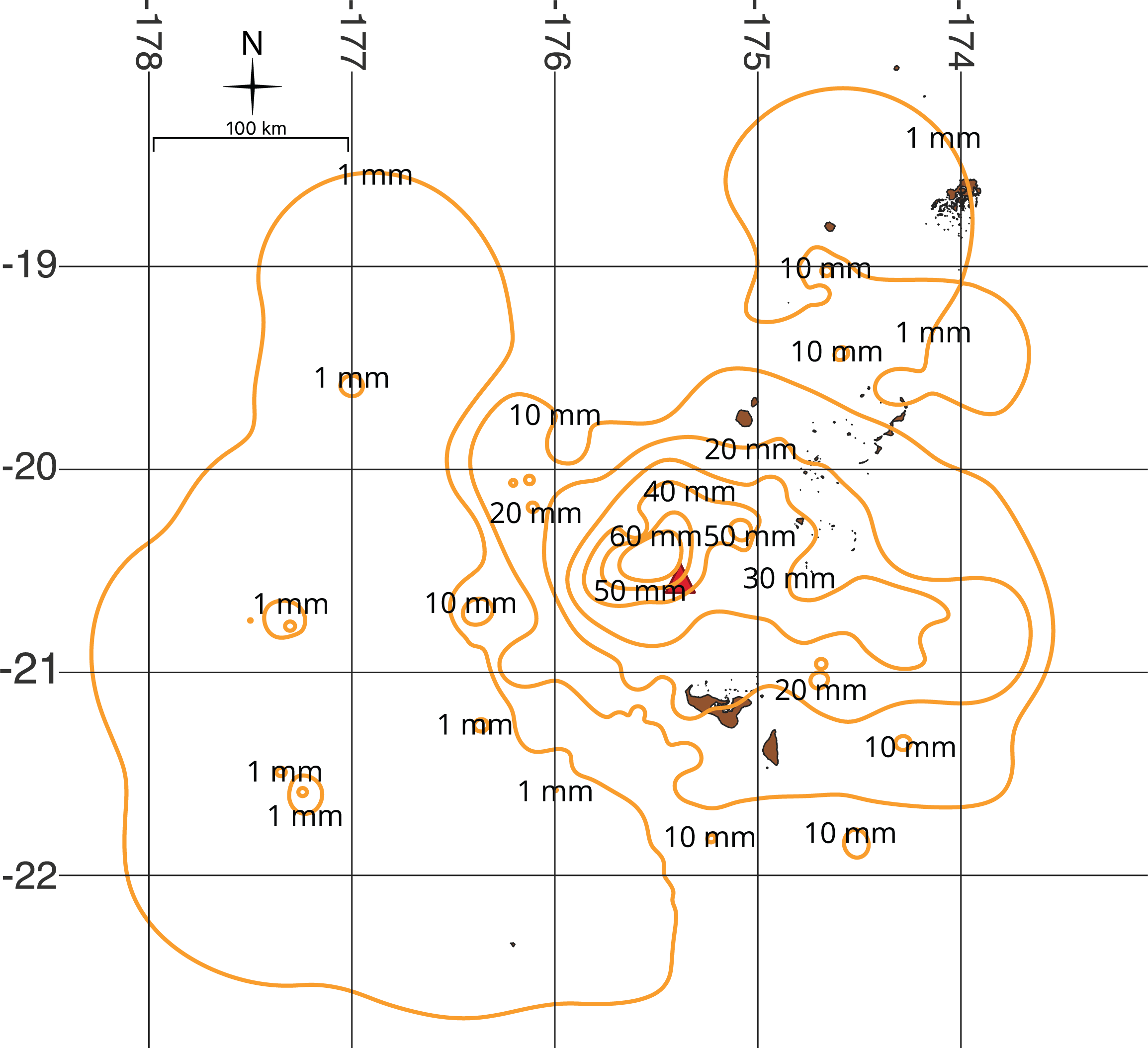


Supplemental Figure 4: Version of Figure 5A from the main text showing only the calculated isopach map from the reflectance/ash image. Islands courtesy of Tonga Department of Statistics and OCHA Office of the Pacific Islands.


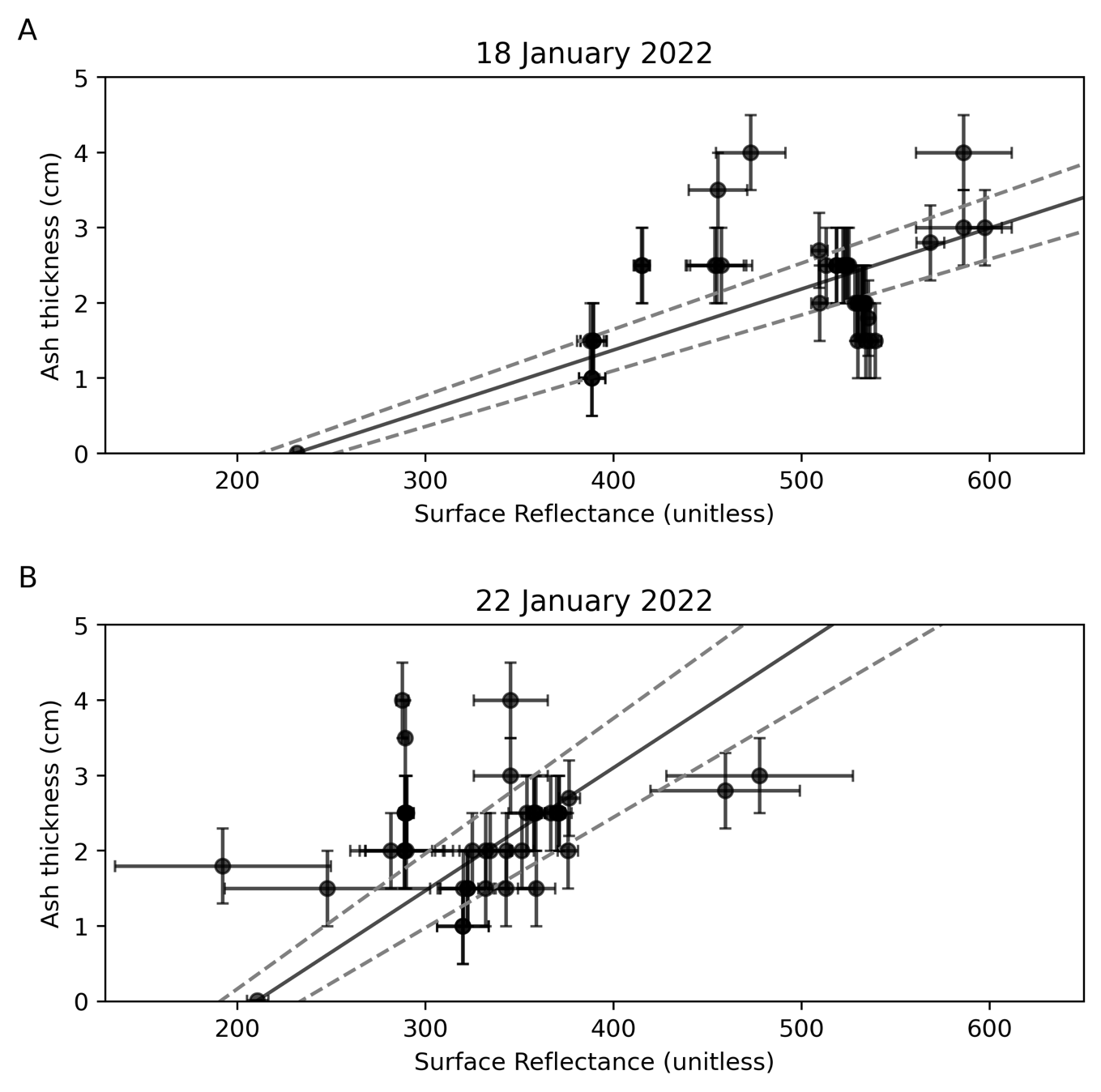


Supplemental Figure 5: Relationship between ash thickness and reflectance with linear fits, from 18 January 2022 (A) and 22 January 2022 (B). 19 January, 20 January, and 21 January are omitted because of sun-glint on the 19^th^ and 21^st^, and missing data not calculated with interpolation of the 0.01 cm ash point on Vava’u on the 20^th^. Procedures for fitting are described in Methods. For (A) R^2^=0.92, (B) R^2^=0.82.


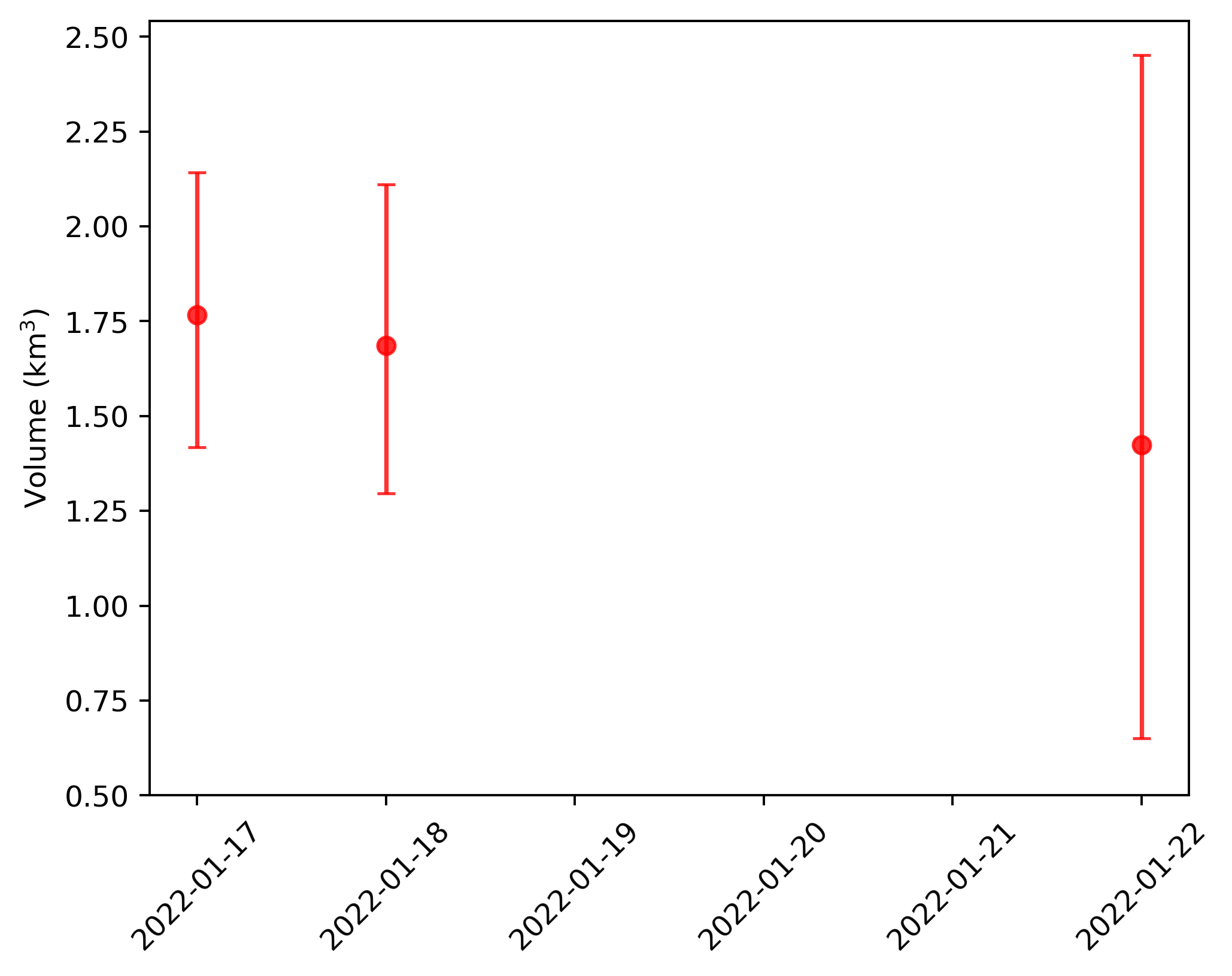


Supplemental Figure 6: Ash volume calculations for different analyzed days. Order of magnitude is preserved, and values are generally consistent. There is an increase in variance as time after the eruption increases.


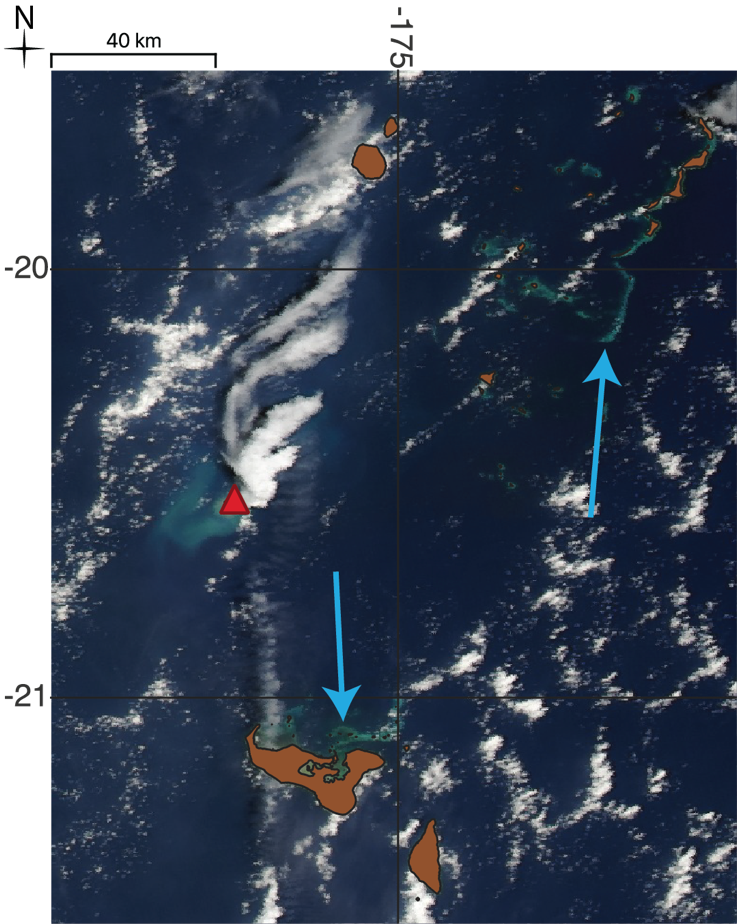


Supplemental Figure 7: Discoloration around islands in the Kingdom of Tonga on 30 December 2021. Blue arrows indicate coastal discoloration. HTHH location is shown with the red triangle. Islands courtesy of Tonga Department of Statistics and OCHA Office of the Pacific Islands.


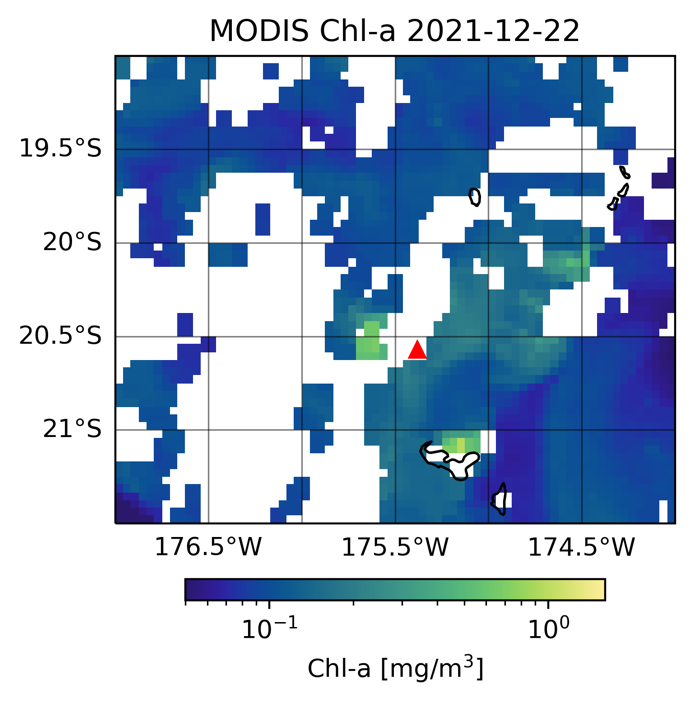


Supplemental Figure 8: Chlorophyll-a estimates from MODIS Aqua satellite. Satellite image is from 22 December 2021, shortly after the 19 December event. Comparison to Figure 3 of (Barone et al., 2022) shows similar values (around 1 mg/m^3^), but the spatial extent of chlorophyll after the January eruption was much larger.
